# Spontaneous Eruption of a Deeply Impacted Premolar After Conservative Treatment of an Associated Dentigerous Cyst: A Case Report

**DOI:** 10.7759/cureus.6414

**Published:** 2019-12-18

**Authors:** Hourya Alnofaie, Omar Alomran, Razan Ababtain, Ahmed Alomar

**Affiliations:** 1 Oral and Maxillofacial Surgery, Princess Nourah Bint Abdulrahman University, Riyadh, SAU; 2 Oral and Maxillofacial Surgery, King Saud University, Riyadh, SAU; 3 Oral and Maxillofacial Surgery, King Abdulaziz Medical City, National Guards Health Affairs, Riyadh, SAU

**Keywords:** dentigerous cyst, marsupialization, impacted tooth, tooth eruption, mental nerve

## Abstract

Dentigerous cysts are benign odontogenic cysts that develop around the crowns of permanent teeth and are considered rare in children. This article reports a case of a 10-year-old girl with an inflammatory dentigerous cyst associated with the right mandibular premolar and deeply displacing it to the lower border of the mandible. To preserve the developing mandibular premolar and the mental nerve, marsupialization of the lesion under local anesthesia was performed. During the first month postoperatively, the impacted premolar naturally started to correct its position towards the normal path of eruption. After 13 months of follow-up, full spontaneous eruption of the impacted premolar with complete resolution of the cystic lesion, new bone formation, and closure of the root apex were observed. The use of marsupialization as a first-line approach is encouraged to treat dentigerous cysts in children, as it has been reported effective in terms of conservative management.

## Introduction

Odontogenic cysts are derived from the odontogenic epithelium, which is derived from the basal epithelium of the stomodeum [1]. Dentigerous cysts (DC) are a type of odontogenic cyst and reported to be the second most common type after radicular cysts, representing 37.9% - 84.5% of all odontogenic cysts [2]. In Saudi Arabia, the prevalence of DC is 25.11%, with a peak incidence between the second and third decades of life and equal oral site distribution [3].

These cysts develop between the enamel epithelium and the enamel of the crown of the associated tooth [4-5]. They are usually asymptomatic and are generally detected by radiographic examination while investigating delayed eruption or swelling [5-6]. Radiographic imaging shows an unerupted tooth surrounded by a unilocular radiolucency with a well-defined sclerotic border [5-6]. They frequently involve third molars, maxillary canines, and premolars but may also involve unerupted supernumerary teeth or odontomas [7]. These cysts may expand the cortical bone, displace, or mobilize the teeth [8].

The conventional treatment of DC is the enucleation and extraction of the involved tooth [9]. However, in cases where the cyst is large, invades vital structures, or tooth preservation is needed, marsupialization can be considered alternatively [10]. The surgical procedure of marsupialization consists of creating a patent cavity through the removal of part of the cystic wall, which allows the contents to drain. This cavity will be irrigated several times until the lining becomes accessible and easy to remove. The offending tooth will usually erupt passively or require surgical and orthodontic intervention [11-12].

We report a case of a DC displacing the mandibular right second premolar and deeply impacting it. Due to the young age of the patient and the necessity to preserve the permanent tooth and mental nerve, the case was conservatively managed with marsupialization.

## Case presentation

A 10-year-old Saudi girl was referred to the oral and maxillofacial surgery department, King Saud University, Saudi Arabia, for the management of a painless swelling in the lower right mandible. Her past medical and dental histories were unremarkable, with no previous or recent habit. The swelling was asymptomatic and did not cause any change in sensation. On examination, a mass was visible over the right mandible extraorally and in the right mandibular buccal vestibule intraorally. The mandibular right second primary molar was crowned and slightly mobile.

**Figure 1:**
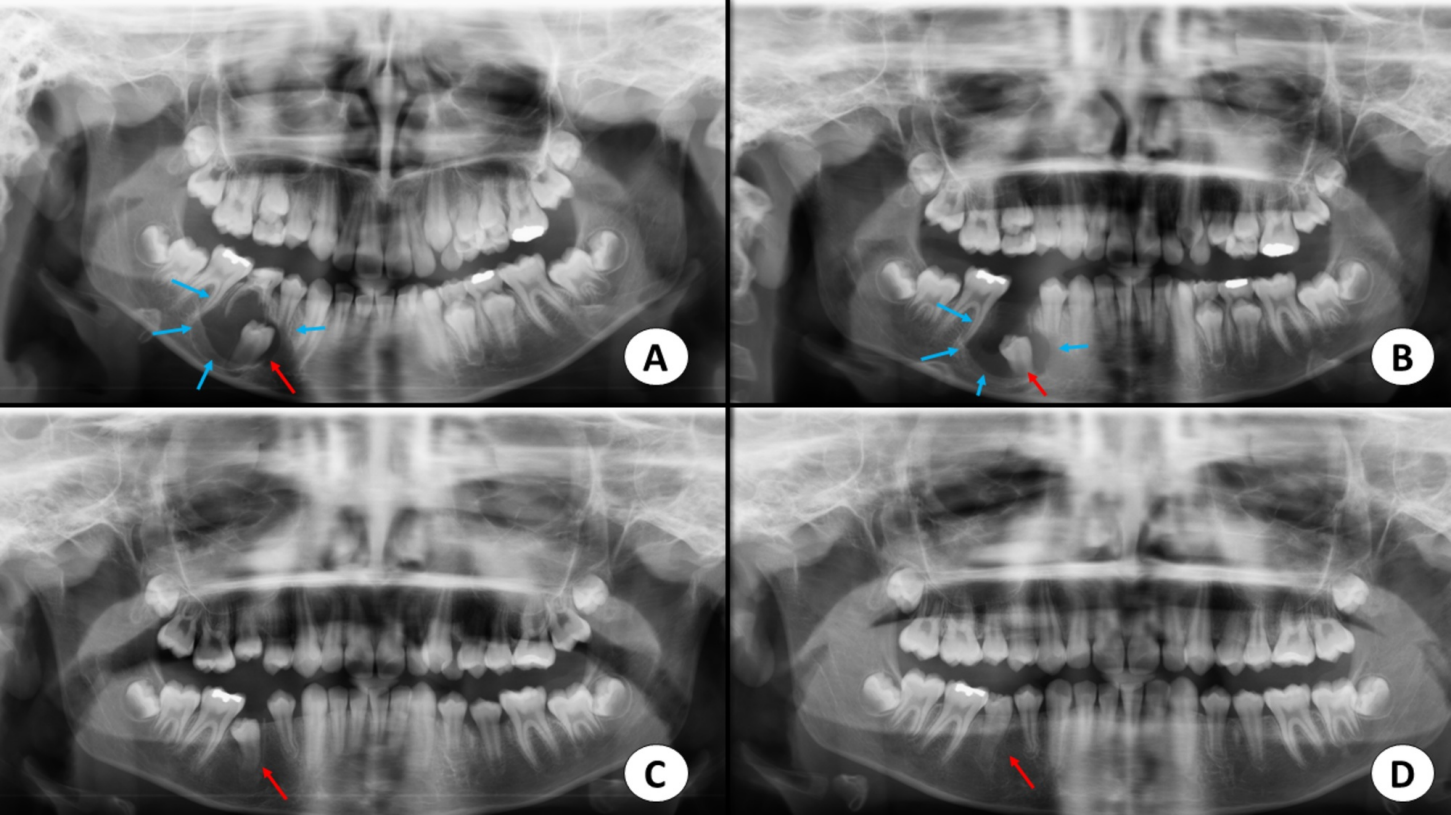
Panoramic radiographs A. Radiograph view demonstrating the dentigerous cyst (blue arrows) related to the unerupted right second premolar (red arrow). B. Postoperative radiographic view after four months, demonstrating the correction of the path of the eruption of the second premolar (red arrow). C. Postoperative view after eight months, displaying the decreased radiolucency around the second premolar and further eruption (red arrow). D. Postoperative view after 13 months showing complete correction of the path of eruption of the right second premolar (red arrow).

A panoramic radiograph revealed a well-defined, radiolucent, osteolytic lesion enclosing the unerupted second premolar beyond the cementoenamel junction (Figure 1A). The lesion was displacing the unerupted second premolar to the lower border of the mandible inferiorly and up to the developing root apex of the erupted first premolar mesially. The lesion measured approximately 3x3 cm in diameter.

Cone-beam computed tomography (CBCT) imaging revealed a well-defined lesion in the mandibular right region surrounding the crown of the unerupted second premolar. The apex of the tooth was still open. The lesion caused expansion and thinning of the buccal wall with no signs of root resorption in the adjacent teeth.

**Figure 2:**
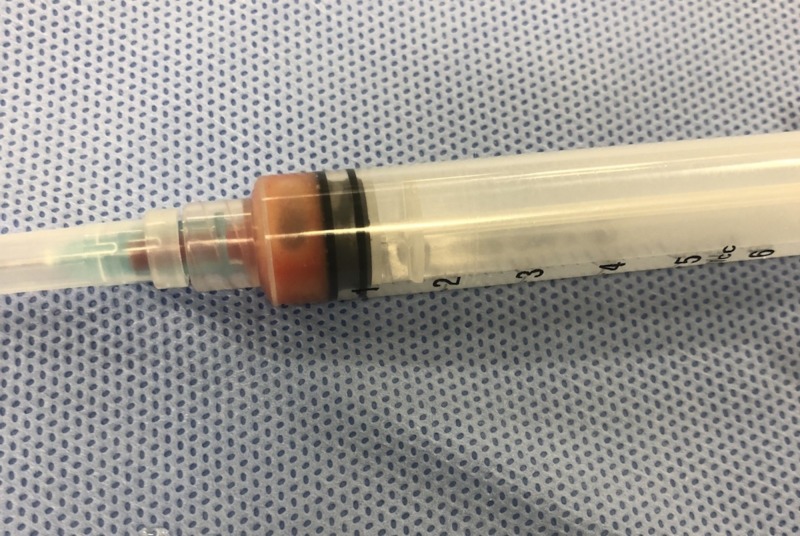
Photograph of the aspirated cystic fluid

The cystic lesion was aspirated and suppurative fluid was evacuated (Figure 2). A provisional diagnosis of an inflammatory DC was made based on the above-mentioned findings.

**Figure 3:**
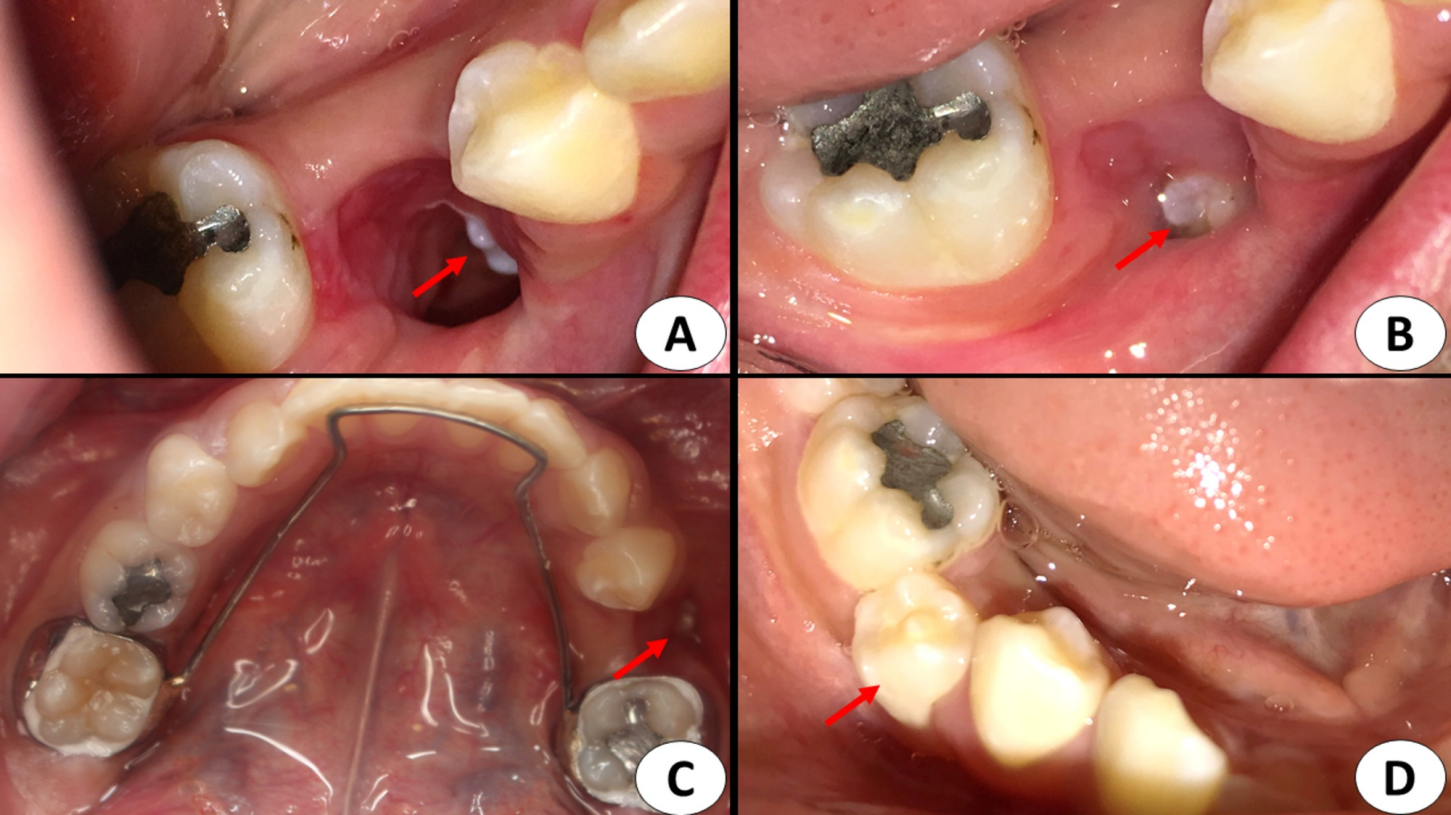
Intraoral photographs A. Marsupialization of the cyst was performed through the extraction socket over the lesion. A small part of the right second premolar can be inspected inside the cavity (red arrow). B. Postoperative view after three weeks where granulation tissues have taken place around the tooth (red arrow). C. Space maintainer cementation to preserve the space for the impacted second premolar (red arrow). D. Postoperative view of the right second premolar, which erupted after 13 months (red arrow).

Under local anesthesia, the deciduous second molar was extracted. A preventive approach was followed to preserve the developing mandibular second premolar. Marsupialization of the lesion was done through the socket of the extracted deciduous second molar. The cystic fluid was evacuated, and the cavity irrigated with normal saline. The impacted tooth was directly inspected (Figure 3A). Lastly, a sterile ribbon gauze was packed inside the cavity.

During the first month postoperatively, the ribbon gauze was changed daily and the cavity irrigated with saline. In the following month, it was changed twice per week. Subsequently, it was changed weekly until complete healing of the cavity was achieved. Moreover, a fixed space maintainer was placed in the patient’s mouth during the first postoperative month, to preserve the space of the second premolar in anticipation of spontaneous eruption (Figure 3C).

Histopathological examination confirmed the definitive diagnosis of an inflammatory DC. A radiograph at the four-month follow-up showed a reduction in radiolucency, with a gradual, spontaneous movement of the tooth into the proper path of the eruption (Figure 1B).

**Figure 4:**
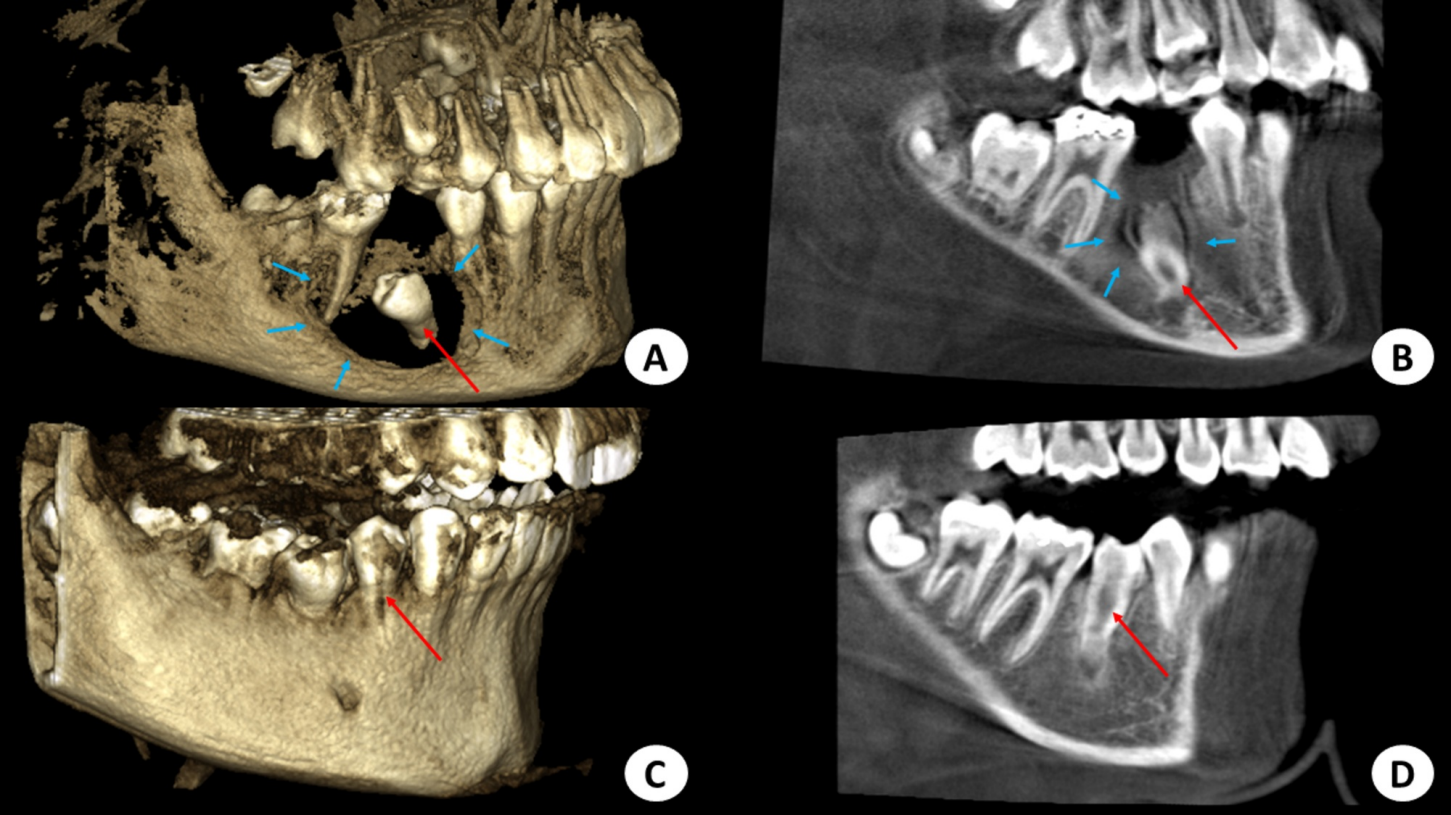
Cone-beam computed tomography images (3D and sagittal views) A. Four-months postoperative 3D view showing the bony defect with surrounding new bone formation (blue arrows) and the initiation of the movement of the second premolar to correct its path of eruption (red arrow). B. A four-month postoperative sagittal view showing correction of the movement of the second premolar (red arrow) and new bone formation (blue arrows). C and D. Postoperative 3D and sagittal views after 13 months where there is optimum new bone growth and complete, spontaneous eruption of the right second premolar (red arrow).

At eight months, there was further movement of the tooth, eruption into the occlusal plane, and subtotal reduction of radiolucency in the follow-up radiograph. Radiographic images showed almost complete ossification of the bony defect and further occlusal movement of the tooth as well as complete root formation (Figure 1C, Figures 4A-4B).

The clinical and radiographic findings after 13 months showed that the second premolar had successfully erupted without orthodontic intervention. (Figure 1D, Figure 3D, Figures 4C-4D) The tooth was vital, with complete root formation and apical closure. The patient will undergo minimal orthodontic alignment of the mandibular second premolar since its eruption is towards the buccal side.

## Discussion

In 1996, Benn and Altini classified DCs into developmental and inflammatory types [12]. Developmental DCs result from impacted mature teeth and mostly affect mandibular third molars [12]. They are usually diagnosed during routine radiographic examination in the late second or third decades of life [12]. The inflammatory type, however, involves a developing permanent tooth and is initiated by an infected necrotic primary tooth that stimulates the immature germ follicle of the permanent affected tooth [12]. Diagnosis of this type is established in the early part of the second decade after suspecting delayed tooth eruption or investigating a swollen mass [12].

The type of DC, in this case, was inflammatory, based on the patient’s young age and features, including a necrotic primary molar and a pus-filled, cystic mass. Marsupialization was chosen since enucleation would sacrifice the permanent second premolar in a young girl and would compromise the surrounding vital structures. Tooth loss at this young age will definitely affect the occlusion, function, and aesthetic appearance. Furthermore, removing the deeply impacted tooth would require significant bone removal, and attempting to enucleate the cyst would place the mental nerve at risk of injury.

According to Pogrel, the decompression of a cyst refers to any technique that relieves the pressure within the cyst that causes it to grow [13]. The difference about decompression is that the patency of the opening must be maintained by placing a tube drain that is sutured to the mucosal margins, while in marsupialization, a pouch is created by suturing the cystic lining directly to the adjacent normal mucosa without any device to allow for direct drainage. The disadvantage with the marsupialization is the need to maintain the patency of the created window. If the window made is small, the epithelium might grow over it and close it entirely [13]. Thus, a larger window must be made in order to avoid that. Nevertheless, creating very large windows can eventually complicate the primary closure, ending up with a mucosal defect [14].

In this case, marsupialization was selected over decompression with a drain as fluids in the deepest inferior surfaces of the cavity are less likely to be completely drained with this method. Moreover, the cystic lining was accessible after removing the deciduous tooth, making a lateral window unnecessary. Despite this, Şahin and Taysi et al. reported successfully decompressing a DC by placing a drain through the superior aspect of the cavity through the extraction socket and replaced it regularly for six months [10,15]. A phenomenon takes place in marsupialization, where a window made in the anterior cystic wall reduces the osmotic pressure within the cavity. This reduces the release of bone-resorbing factors, which, in turn, facilitates bone healing and remodeling [15].

Abu-Mostafa and Abbasi used a similar approach of marsupialization, but they applied fusidic acid cream to the gauze and intervened orthodontically [16]. A different approach through compression was followed by Bozdogan et al. and Qian et al., where they utilized a lateral window, which can result in either a larger bone defect, ectopic eruption, or malocclusion of the developing permanent teeth [17-18].

Despite DCs inhibiting the eruption of associated permanent teeth, the development of the roots of these teeth continues [19]. In this case, the impacted tooth succeeded to spontaneously erupt, with complete root formation. Moreover, the vitality of the tooth was not affected, demonstrating the benefit of this conservative technique for such cases.

Features that distinguish this case from others published in the literature are the depth of impaction and the ability of the affected tooth to not only spontaneously erupt but to also correct its path of eruption and travel from a deep point distally then superiorly, to erupt normally in the oral cavity, with no need for orthodontic extrusion [11]. According to Hyomoto et al., the shrinkage of the cyst can encourage tooth eruption, which usually happens during the first three months after marsupialization [20]. Therefore, patients should be observed closely during this period before attempting the enucleation or extraction of the offending teeth.

The management approach, in this case, was clear, simple, and conservative. A long follow-up period during treatment, along with close observation and optimal compliance were important parts of the management.

Since the performed procedure did not have any involvement with trying a new experiment, procedure, material, or medication on the patient and only demonstrated performing a well-known surgical procedure but with a pathology that is usually known to respond best to a more aggressive treatment, no Institutional Review Board (IRB) approval was required according to the IRB of King Saud University, where the procedure was performed.

## Conclusions

The results of the current case suggest that mandibular premolars associated with DCs can erupt spontaneously after marsupialization in preadolescents. Since immature teeth with incomplete root formation and open apices have optimal eruption potential, and children have greater vascularity and bone regeneration than adults, the efficacy of conservative surgical management in young patients is usually high. Thus, marsupialization is recommended to be the first line of treatment in the conservative surgical management of DCs in children.
